# Enhanced multi view 3D reconstruction with improved MVSNet

**DOI:** 10.1038/s41598-024-64805-y

**Published:** 2024-06-19

**Authors:** Guangchen Li, Kefeng Li, Guangyuan Zhang, Zhenfang Zhu, Peng Wang, Zhenfei Wang, Chen Fu

**Affiliations:** 1 Shandong Jiaotong University, Haitang Road 5001, Jinan, 250357 China; 2 Shandong Zhengyuan Yeda Environmental Technology Co., Ltd., Jinan, 250101 China

**Keywords:** Engineering, Computer science

## Abstract

Although 3D reconstruction has been widely used in many fields as a key component of environment perception, existing technologies still have the potential for further improvement in 3D scene reconstruction. We propose an improved reconstruction algorithm based on the MVSNet network architecture. To glean richer pixel details from images, we suggest deploying a DE module integrated with a residual framework, which supplants the prevailing feature extraction mechanism. The DE module uses ECA-Net and dilated convolution to expand the receptive field range, performing feature splicing and fusion through the residual structure to retain the global information of the original image. Moreover, harnessing attention mechanisms refines the 3D cost volume's regularization process, bolstering the integration of information across multi-scale feature volumes, consequently enhancing depth estimation precision. When assessed our model using the DTU dataset, findings highlight the network's 3D reconstruction scoring a completeness (comp) of 0.411 mm and an overall quality of 0.418 mm. This performance is higher than that of traditional methods and other deep learning-based methods. Additionally, the visual representation of the point cloud model exhibits marked advancements. Trials on the Blended MVS dataset signify that our network exhibits commendable generalization prowess.

## Introduction

In recent years, 3D reconstruction technology has been widely used in various fields. 3D reconstruction technology converts real scenes into mathematical point clouds in line with computer logic. This process involves depth data acquisition, preprocessing, point cloud registration and fusion, and surface generation. 3D reconstruction technology has key applications in virtual reality, automated driving, medical imaging, etc. Multi-view 3D reconstruction (MVS) is one of the core research directions, and the MVS algorithm is a 3D reconstruction method based on multiple views of an object and has a high computational cost. Mainly, it infers three-dimensional information through an image and uses different viewpoints from two or more images to restore this three-dimensional data. With a series of calibrated multi-views and standard camera parameters, the geometric relationship between transformations is established, such as through homography matrix transformations for different views. Subsequently, the algorithm determines the depth of each view and ultimately fuses these to reconstruct a dense point cloud, restoring the three-dimensional scene. Traditional multi-view 3D reconstruction algorithms^1–3^ use artificially designed features for feature extraction of image targets and use them for 3D reconstruction. But in sparsely textured, non-diffusely reflective scenes, feature extraction is difficult, and 3D reconstruction results are poor. In recent years, deep learning has provided a new approach to this task. More and more researchers have started to use deep neural networks to automatically extract image features. And the extracted features are more semantic^4^, which leads to a more stable matching, and the similarity between views^5–7^ and regularization^8–10^ can be automatically computed through the deep learning approach, which can be adapted to most of the scenes. Yao et al.^11^ proposed a deep learning based multi-view stereo network (MVSNet), which pioneered the embedding of the differentiable homography transformation into the neural network, and combined the camera model transform with the neural network to realize the end-to-end multi-view 3D reconstruction. In addition, MVSNet adopts a variance-based feature mapping method to avoid the decrease in matching cost due to the increase in the number of images. A U-Net-like 3D convolutional network and regression operation are employed to finally realize the depth estimation of the reference image and outperform the traditional algorithms in several aspects, such as reconstruction accuracy and running time. However, as the number of depth samples increases, MVSNet occupies a large amount of computational resources. In order to solve the problem of consuming too much memory when training the model of MVSNet, the author Yao proposed a new network R-MVSNet^12^ based on MVSNet, which alleviates the memory consumption and thus makes large-scale 3D reconstruction possible. Luo et al.^13^ proposed a point multi-view stereo network (P-MVSNet) based on the region matching confidence cost volumes. Gu et al.^14^ used a cascade structure for constructing the cost volumes, first estimating the coarser cost volumes, and then further improving the image resolution to obtain a higher resolution and higher accuracy depth map. A series of 3D reconstruction methods based on deep learning have shown excellent reconstruction performance. We recognize that although existing methods have made significant progress, there remains a vast scope for deeper feature learning and model optimization in the field of 3D reconstruction. Therefore, based on the MVSNet model we propose the DEC-MVSNet.

Our main contributions are as follows:

1. Proposed a novel network structure based on MVSNet, which is able to handle image data with different scales and different resolutions, and with better reconstruction results.
2. We propose to use the DE module combined with the residual structure as the feature extraction part of the model, which increases the sensory field of the network and enables the network to efficiently extract richer pixel information in the image.
3. We introduce the adaptive attention CBAM module between every two cost volumes and probabilistic volumes of the same scale in MVSNet. Through adaptive learning of channel and spatial attention weights, this module emphasizes important features and diminishes less relevant ones, thereby enhancing the accuracy of depth estimation and subsequently improving the 3D reconstruction results.

## Materials and methods

### Overall network architecture

**Figure 1:**
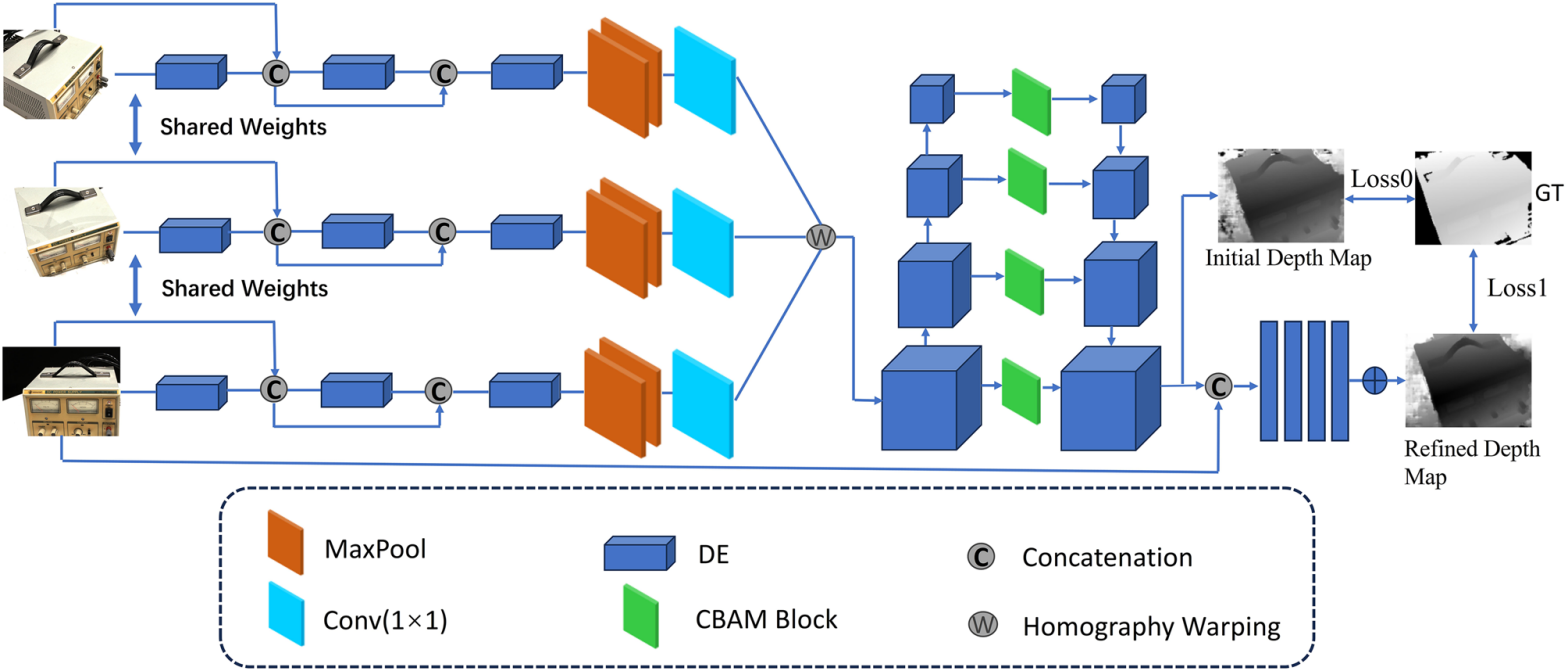
The network design of DEC-MVSNet. Input images first pass through a 2D feature extraction network and differentiable homography warping to generate the cost volume. The final depth map is regressed from the regularized probability volume and refined using the reference image.

We propose the DEC-MVSNet network architecture based on the foundation of MVSNet, which mainly consists of four major components: feature extraction, differentiable homography transformation, cost volume regularization, and depth map optimization. The overall network structure is shown in Fig. 1.

### Feature extraction

**Figure 2:**
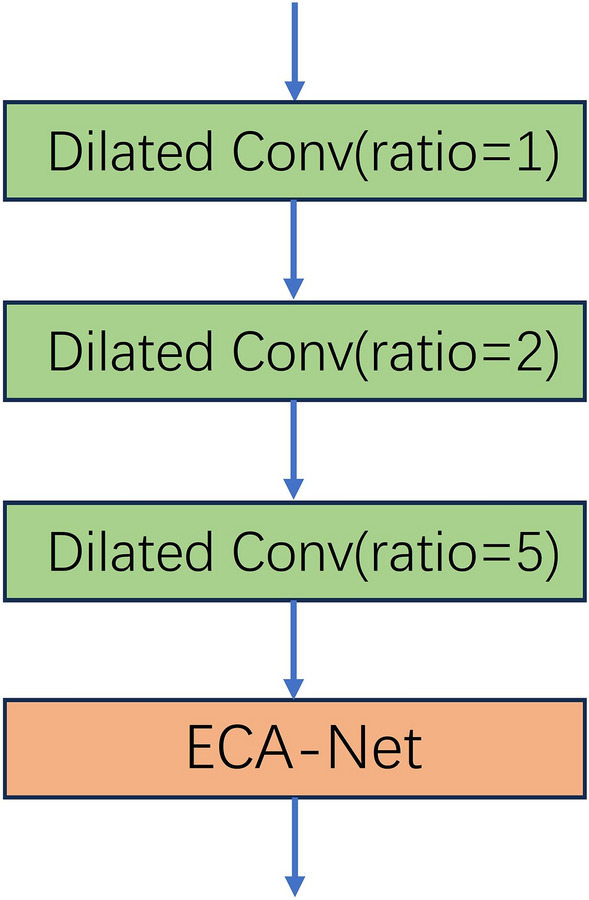
Structure diagram of the DE module, showing its configuration in DEC-MVSNet. Its main function is feature extraction.

This section focuses on the feature extraction part of the network. It primarily extracts features from the input image to capture pixel information. Existing deep learning methods primarily target indoor scenes or specific objects, integrating feature information through multiple views and then generating cost volumes based on homography transformations. Many of these algorithms enhance the overall integrity of the scene but sacrifice the integrity of the local texture. The reason for this phenomenon is that the role of the feature extraction network is neglected, leading to the loss of detail information in the source view after the homography transformation. In order to obtain more image semantic information, we use the DE module combined with the residual structure to replace the original MVSNet feature extraction part. The DE module is shown in Fig. 2. It is mainly composed of dilation convolution and ECA-Net. The dilation convolution means that compared with the ordinary convolution, in addition to the size of the convolution kernel, there is a dilation rate parameter, which is mainly used to indicate the size of the dilation. Dilated convolution can expand the receptive field without increasing the size of the convolution kernel, allowing for a larger feature scope. The formula for calculating the size of the receptive field after the convolution kernel is processed by dilation convolution is Eq. (1), where n is the receptive field after dilation convolution, k is the original convolution kernel size, and r is the dilation rate.

1 $$n=r\times k+\left(r-1\right)$$

**Figure 3:**
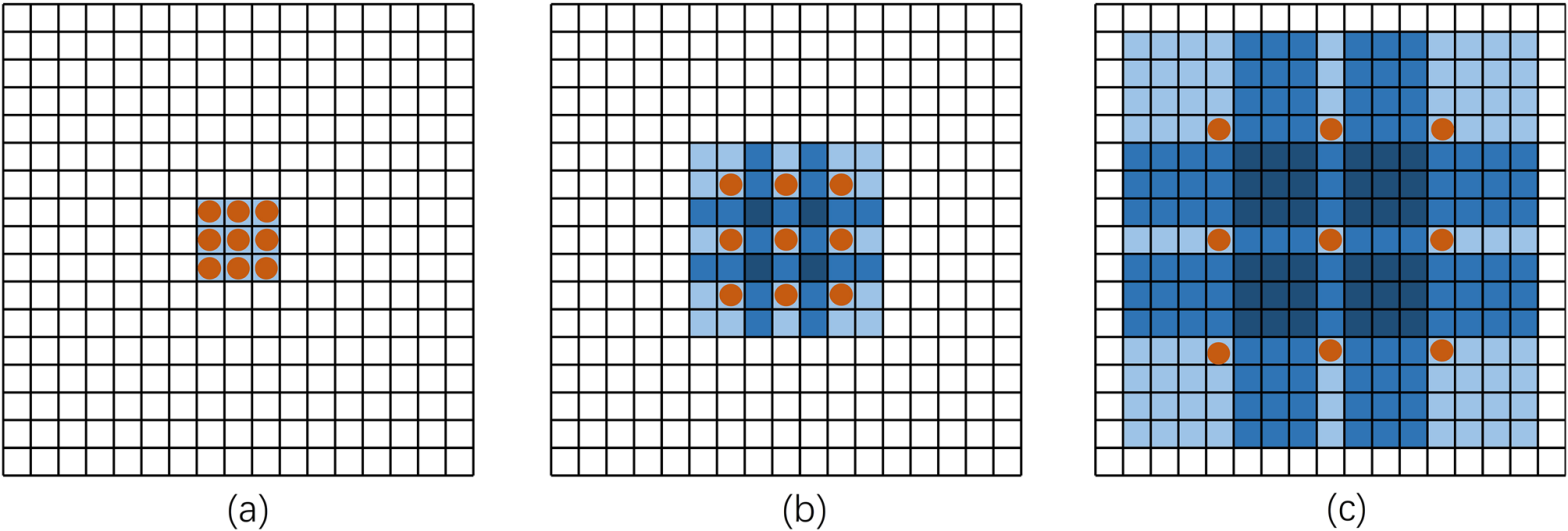
The expansion effect of the receptive field: (**a**) is a receptive field with an expansion rate of 1, (**b**) is a receptive field with an expansion rate of 2, and (**c**) is a receptive field with an expansion rate of 4.

The effect of receptive field expansion is illustrated in Fig. 3, where the convolution kernel is the same and the size is 3 × 3, but the receptive fields are different. The corresponding receptive fields are 3 × 3, 7 × 7, and 15 × 15 when the dilation rate is 1, 2, and 4, respectively.

The gridding effect^15^ inevitably occurs when using the dilated convolution. The dilated convolution's kernel covers an effective region that forms a grid shape when sliding, causing loss of information at neighboring points; this issue worsens with larger dilation rates. In order to solve the grid effect on feature extraction, this paper introduces the design concept of HDC^16^, and follows the HDC^16^ principle when designing the dilated convolution. The dilated convolution designed in this paper satisfies the following characteristics (1)The dilation rate values between different dilated convolution layers vary in a pattern resembling a sawtooth wave; (2)There cannot be a common divisor other than 1 between the stacked convolutional layer expansion rates; (3) The dilation rate needs to be selected according to Eq. (2). Where: $r_{i}$ is the dilation rate of layer ${\text{i}}$;$M_{i}$ is the maximum dilation rate of layer ${\text{i}}$.

2 $$M_{i} = \max [M_{i} + 1 - 2r_{i} ,M_{i} + 1 - 2(M_{i} + 1 - r_{i} ),r_{i} ]$$

To summarize the above conditions, the dilation rate of each dilated convolution block selected in this paper is 1, 2 and 5, respectively, and the number of convolution kernels is set to 3. The dilated convolution model designed in this paper can rapidly expand the receptive field of the convolution kernels through the increase of the dilation rate to obtain multi-scale information and improve the performance of the model.

**Figure 4:**
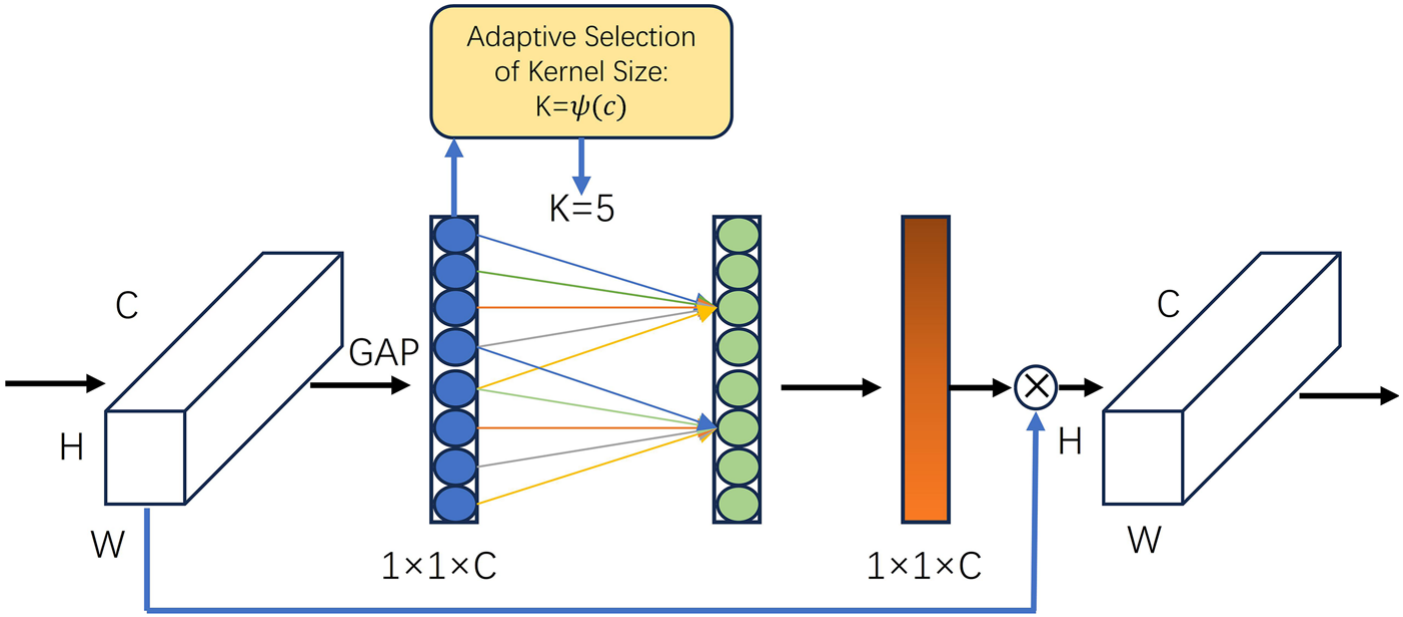
Overview of the ECA-Net architecture, highlighting its efficient channel attention mechanisms for feature enhancement.

Attention mechanism has been proven to be a potential method to enhance deep convolutional neural networks, which has achieved very good results in image classification and recognition, target detection, and other computer vision aspects. The principle of the attention mechanism is based on weight coefficients, reweighting, and summing. Its essence lies in the ability to match features based on the inputs for different tasks. The ECA-Net attention mechanism efficiently reduces the amount of parameter calculation and improves the detection speed. Its working principle is shown in Fig. 4. It is an attention mechanism that can effectively capture cross-channel interactive information. After channel-by-channel global average pooling, local cross-channel information interactions are captured by considering each channel and its k nearest neighbors. This approach ensures both efficiency and effectiveness. The ECA-Net module can be efficiently implemented by a fast one-dimensional convolution of size k, where the kernel size k represents the coverage of local cross-channel interactions, i.e., how many immediate neighbors are involved in the attentional prediction of a channel. k is determined by an adaptive method, where the coverage of interactions (i.e., kernel size k) is proportional to the number of channel dimensions. ECA-Net effectively implements local cross-channel interaction strategies through one-dimensional convolution and requires no dimensionality reduction. This module contains only a small number of parameters, which can lead to significant performance improvements. In this paper, the ECA-Net module is added to the feature extraction network, and the image is accordingly subjected to dilated convolution with dilatation rates of 1, 2, and 5 before passing through this attention module.

### Differentiable homography transformation

The key role of differentiable homography transformation is to project the N feature maps acquired in the preceding step onto multiple parallel planes beneath the reference image, resulting in N feature volumes. MVSNet employs an algorithm akin to plane scanning to compute matching costs^17^. Post-feature extraction, every image yields its respective feature map. Next, using the primary optical axis direction of the reference view as the scanning direction. The primary optical axis ahead of the reference view spans a depth range from $\theta_{min}$ to $\theta_{max}$, with an interval of $\theta_{scale}$. This approach results in a camera cone with varying depths. However, projecting images onto positions of distinct depths leads to variations in image sizes. Hence, interpolation ensures uniform dimensions for each projected image. A cost volume is derived through the application of differentiable homography transformation, guaranteeing end-to-end training within deep learning paradigms.

### Cost volume regularization

**Figure 5:**
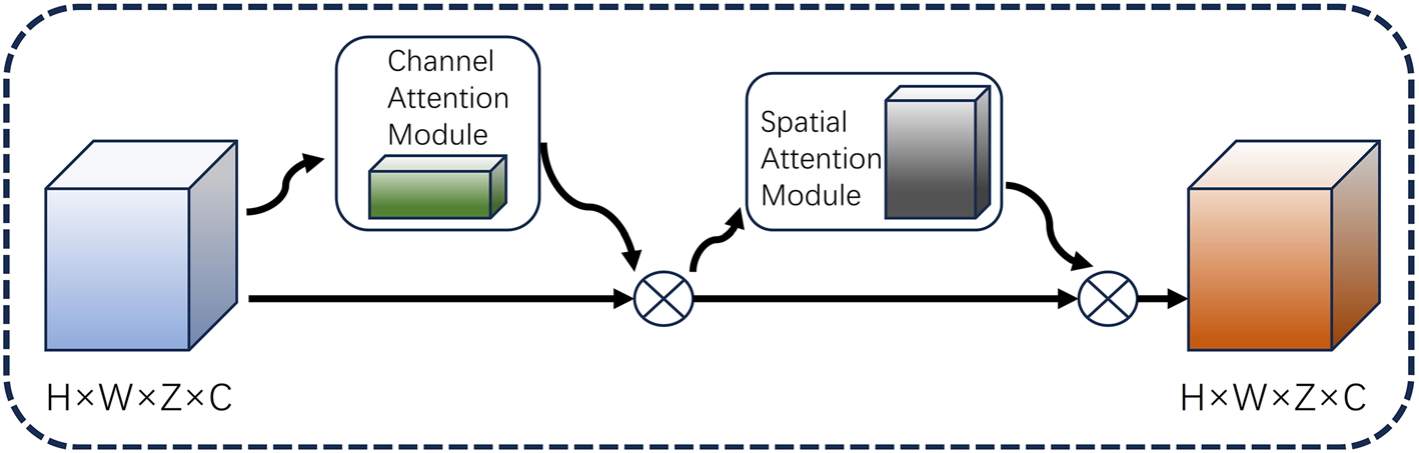
CBAM structure diagram.

The feature body computed from the image features may be affected by noise due to non-Lambertian surfaces or occlusion, in order to eliminate or minimize the effect of this noise. We need to perform a smoothing operation on the cost volume after the step of generating the cost volume is completed, and then go through the multiscale 3D attentional convolutional neural network to output the probabilistic body $P$. Probabilistic maps measure the likelihood of image pixels at different values of depth size. After the probability body is generated, the probability values are normalized in the dimension of depth using the softmax operation. For any point $(x,y,d)$ on the probability body $P$, its corresponding value indicates that the probability that pixel $(x,y)$ on the 2D image is at depth $d$ is ${P}_{\left(x,y,d\right)}$. The generated probability body can be well used for depth value prediction, not only for pixel-by-pixel depth prediction, but also for measuring the confidence level of the estimated depth. Unlike MVSNet, the DEC-MVSNet network structure incorporates the CBAM attention mechanism. Irrelevant features are suppressed by way of learning the channel weights, which improves the representation performance of the convolutional features and yields more accurate probabilistic bodies. The structure of the CBAM module is shown in Fig. 5, which sets the attention in two dimensions, i.e., Channel Attention Module (CAM) and Spatial Attention Module (SAM), respectively.

(1) Channel Attention Module

**Figure 6:**
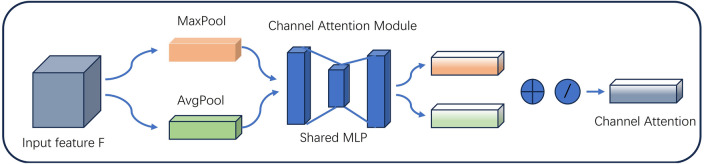
CAM structure diagram.

The channel attention module is shown in Fig. 6, which performs maximum pooling and average pooling on the input feature map F, respectively, captures the maximum and average information in each channel, Then passes the resulting 2 feature maps to the multilayer perceptron, where the 1st fully connected layer is downscaled to reduce the computational complexity, and the 2nd fully connected layer is upscaled, which determine the channel weights by learning the relationship between the channels. The two features after the multilayer perceptron are summed to generate the channel weights by the sigmoid activation function, and then these weights are multiplied with the input features to adjust the features of each channel accordingly to generate the input features needed by the spatial attention module. The specific formula is as follows: Eq. (3).

3 $$M_{{\text{C}}} ({\text{N}}) = \sigma ({\text{MLP}}({\text{AvgPool}}({\text{N}})) + {\text{MLP}}({\text{MaxPool}}({\text{N}})))$$

where $\sigma$ is the sigmoid function; MLP is the multilayer perceptron; ${\text{AvgPool}}({\text{N}})$ and ${\text{MaxPool}}({\text{N}})$ denote average and max pooling, respectively; and $M_{{\text{C}}} ({\text{N}})$ is the channel attention module.

(2) Spatial Attention Module

**Figure 7:**
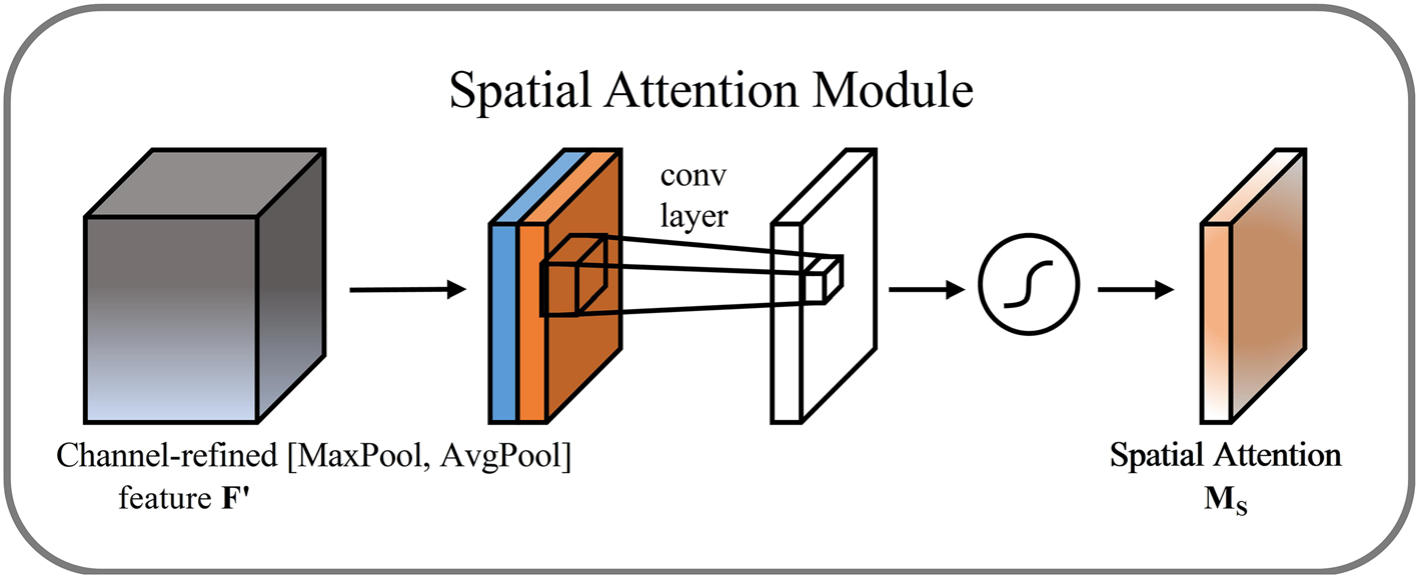
SAM structure diagram.

The spatial attention module is shown in Fig. 7. The channel attention module's output feature map serves as the input for this module, and after maximum pooling and average pooling, the results of the two are combined to form a feature map with two channels. The merged feature map is transformed into a single-channel map through a convolutional operation. Spatial weights are generated after sigmoid activation function, and these weights will be multiplied with the input feature map to weight the feature response of each spatial location to get the final feature map. The specific calculation formula is as follows: Eq. (4).

4 $$M_{{\text{s}}} ({\text{n}}) = \sigma (f([{\text{AvgPool}}({\text{n}}),{\text{MaxPool}}({\text{n}})]))$$

where: n is the input feature map; $f$ is the 7 × 7 convolution operation; ${\text{AvgPool}}({\text{n}})$ and ${\text{MaxPool}}({\text{n}})$ denote average and max pooling, respectively; and $M_{{\text{s}}} ({\text{n}})$ is the spatial attention module.

### Depth map estimation and optimization

After obtaining the probability distributions of the image pixels at different depths, the depth map can be estimated based on these probability distributions. The simplest method to obtain the depth map is to take the depth value with the highest probability for each pixel across different depths, which corresponds to the argmax operation. Although this method is simple and straightforward, the depth map estimated in this way is not accurate enough. Moreover, since argmax is not differentiable, it is not possible to learn the parameters of the network by backpropagation during training. In order to solve the above problems, we use soft argmin for depth value estimation with the following formula: Eq. (5).

5 $$D = \mathop \sum \limits_{{d = d_{\min } }}^{{d = d_{\max } }} d \times P(d)$$

This procedure primarily involves computing the weighted average of all probabilities. Specifically, $d_{\min }$ denotes the minimum depth value, while $d_{\max }$ signifies the maximum depth value. $d$ stands for the predetermined depth sampling value, $P(d)$ with representing the likelihood of a pixel's presence at depth $d$. This operation is fully differentiable, allowing errors to be back propagated and optimized during network training. Although the final depth map obtained from the probabilistic body is a high-quality depth map, it is possible that the boundaries of the depth map are not very clear, so the depth map needs to be optimized. We sum the reference image with the initial depth map returned by the network at the end to get a 4-channel input, and set up a refinement network at the end of the network, which contains three 32-channel 2D convolutions and a 1-channel convolutional layer for residual learning. After processing through this network, the 4-channel input is transformed into a refined depth map.

### Loss function

The loss function is designed to take into account both the initial and optimized depth map losses. The network uses the average absolute error between the true depth map and the estimated depth map as the training loss. Since the ground truth (GT) depth map is mostly incomplete, only pixels with valid GT labels are considered in this method, and the loss function is shown in Eq. (6):

6 $${\text{Loss }} = \sum\limits_{{p \in P_{{\text{valid }}} }} {\underbrace {{\left\| {d(p) - \hat{d}_{i} (p)} \right\|_{1} }}_{{{\text{Loss }}1}}} + \, \lambda \cdot \underbrace {{\left\| {d(p) - \hat{d}_{r} (p)} \right\|_{1} }}_{{{\text{Loss }}2}}$$

where $P_{{\text{valid }}}$ is the set of valid pixels in the true value, $d(p)$ is the true depth value of pixel $p$, $\hat{d}_{i} (p)$ is the depth value of pixel $p$ of the initial depth map, $\hat{d}_{r} (p)$ is the depth value of pixel $p$ of the optimized depth map, $\lambda$ is the weighting coefficient, the larger $\lambda$ means that ${\text{Loss }}2$ is more important, and the parameter $\lambda$ is set to 1.0 in the experiment, ${\text{Loss }}1$ denotes the loss between the true value and the initial depth map, and ${\text{Loss }}2$ denotes the loss between the true value and the optimized depth map.

## Experiments and results

### Datasets

The main dataset for this experiment comes from the DTU dataset^18^, which includes 124 scenes of small indoor objects or settings specifically captured for multi-view stereo matching. Each of the images, sized 640 × 512, was taken under seven different lighting conditions. Accurate structured light scanning was also performed for reference and evaluation.

The additional dataset is the large-scale scene dataset BlendedMVS^19^, this dataset has more than 17 k high-quality training samples covering 113 different reconstructed scenes including buildings, streetscapes, sculptures, and small objects, and there are 20–1000 input images for each scene, each with the size of 768 × 576, totalling 17,818 images. The details of the datasets used for the experiment are presented in Table 1.

**Table 1:** Details of the DTU and BlendedMVS datasets used in our experiments, including number of images.

Datasets	Training	Validation	Number of images
DTU	27,097	7546	42,189
BlendedMVS	17,818	5618	23,436

### Experimental setup and evaluation metric

The experimental environment for this chapter is as follows, all experiments were conducted using pytorch 1.10.0 and python 3.8 on an Ubuntu 20.04 operating system, with an RTXA5000 graphics card boasting 24 GB of VRAM. The Adam optimizer was used for the experiments. The batch size was set to 2. The initial learning rate was 0.001. Due to the slow decrease in loss during the later stages of training, we adopted a dynamic learning rate adjustment strategy. Specifically, the learning rate was halved at the 10th, 12th, and 14th training epochs.

For quantitative evaluation, we calculate the accuracy and the completeness of the distance metric^18^. The matlab code for the distance metric is provided by the DTU dataset. Ideally, the algorithm should have both high accuracy and high completeness, but these two metrics have a mutually constraining relationship. Therefore, in order to conduct a fairer evaluation analysis, both Accuracy (Acc) and Completeness (Comp) are considered. Overall is used to denote the comprehensive score. It is calculated as the average of Accuracy (Acc) and Completeness (Comp). Ultimately, the comprehensive performance of the algorithm is measured based on the average Overall to evaluate the overall reconstruction quality.

### Experimental analysis on the DTU dataset

#### Model training and testing

In the training phase, we set the number of input images N = 3 (1 reference image, 2 neighboring images of the reference image), and the resolution of the image is set W = 640, H = 512. In the testing phase, the number of input images is set N = 5 (1 reference image, 4 neighboring images of the reference image). When selecting the input of viewpoint image, the score obtained for each reference image and neighboring images is calculated according to Eq. (7), and the input of viewpoint image is selected based on the result of the score.

7 $$s(i,j) = {\Sigma }_{p} G(\theta_{ij} (p))$$

where $p$ is the common trajectory of image $i$ and image $j$. $\theta_{ij} (p)$ represents the angle at which $p$ offsets the baseline, and the computation of $\theta_{ij} (p)$ is shown in Eq. (8). $G$ is a Gaussian function, which is computed differently depending on $\theta$. The computation of the Gaussian function is shown in Eq. (9). Where $c$ is the center of the camera.

8 $$\theta_{ij} (p) = (\begin{array}{*{20}c} {180/\pi )\arccos (\begin{array}{*{20}c} {(c_{i} - p)(c_{j} - p)} \\ \end{array} )} \\ \end{array}$$

9 $$G(\theta ) = \left\{ {\begin{array}{*{20}l} {\exp \left( { - \frac{{\left( {\theta - \theta_{0} } \right)^{2} }}{{2\sigma_{2}^{2} }}} \right),\theta > \theta_{0} } \hfill \\ {\exp \left( { - \frac{{\left( {\theta - \theta_{0} } \right)^{2} }}{{2\sigma_{1}^{2} }}} \right),\theta \le \theta_{0} } \hfill \\ \end{array} } \right.$$

In the experiment, parameters $\theta$, ${\upsigma }_{1}$ and ${\upsigma }_{2}$ were set to 5, 1 and 10, respectively.

**Figure 8:**
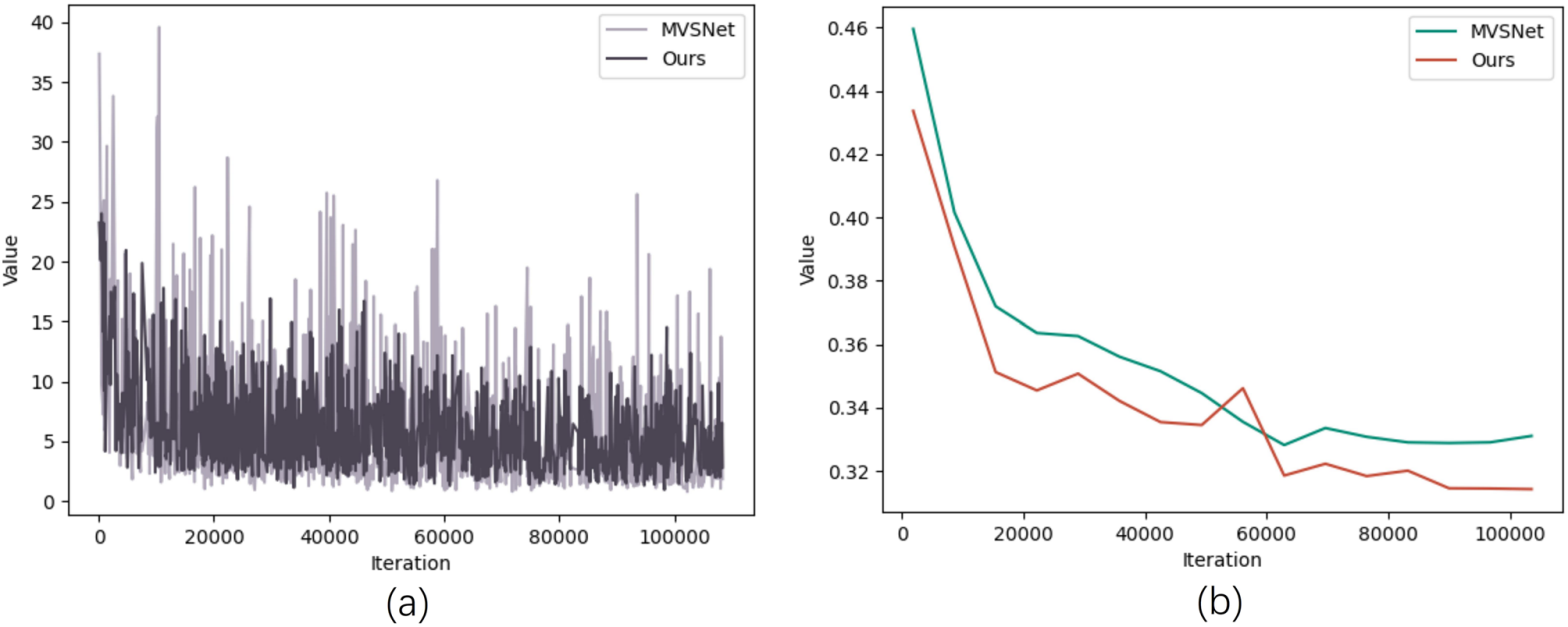
Graphs showing the progression of training loss and the corresponding error rate on the DTU dataset: (**a**) is the training Loss, (**b**) is the error rate in DTU.

Finally, use the DTU to train this model and MVSNet in the same environment. As shown in Fig. 8, observing the Loss curve (a) and Error rate curve (b) after the network training, it can be found that our model Loss curve and Error rate curve have obvious advantages, which indicates that the performance of our improved model is better.

#### Depth map fusion

**Figure 9:**
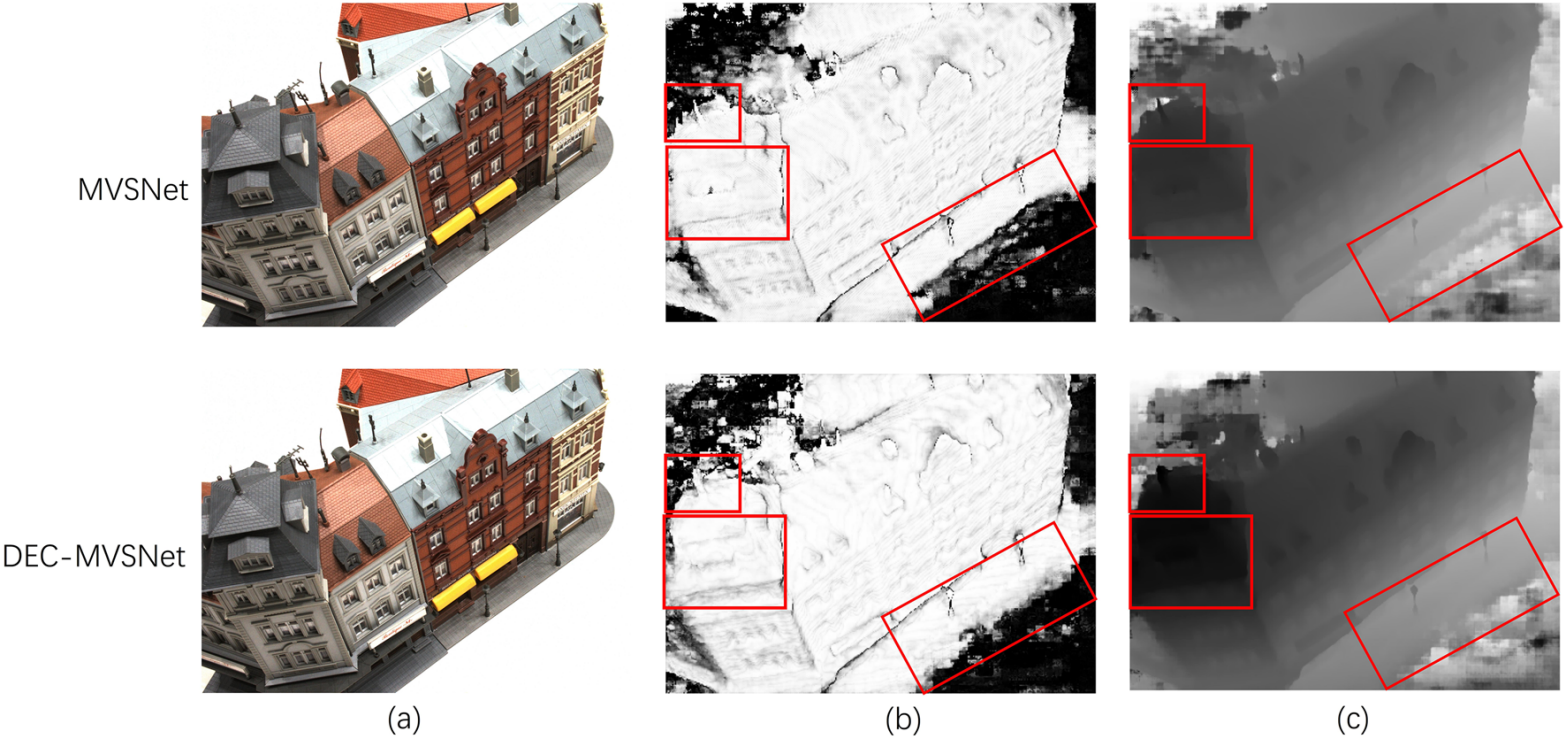
Comparison of Scan9 probability map and depth map estimates in the DTU dataset: (**a**) is the reference image, (**b**) is the probability and (**c**) is the depth map, the red box highlights the difference between the comparison images.

The probability map undergoes depth fusion to produce the depth map. Furthermore, all depth images in the DTU test set are fused using Galliani's method^20^. By proposing the incorporated CBAM attention mechanism, optimization is performed in the cost volume regularization part to get more accurate probability maps, thus predicting more accurate and complete depth maps. The experimental results are shown in Fig. 9.

#### Ablation study

In order to verify the effectiveness of our improved module, we carry out ablation experiments to validate the DE module and CBAM in DEC-MVSNet, √ in the table means the module has been added, and × means the module has not been added. It can be seen that after the improvement of the feature extraction network and the regularization part of the cost volume regularization, there is a noticeable improvement over the baseline MVSNet model in terms of both Comp and Overall metrics, which proves the correctness of the direction of this improvement after the experiments. In the quantitative results are shown in Table 2.

**Table 2:** Results of ablation studies on the DTU dataset, showing the impact of various network components on performance metrics using the distance metric (lower is better).

DE	CBAM	ACC	Comp	Overall
×	×	**0.396**	0.527	0.462
√	×	0.431	0.415	0.423
√	√	0.425	**0.411**	**0.418**

Significant values are given in bold.

### Comparative experiments

**Figure 10:**
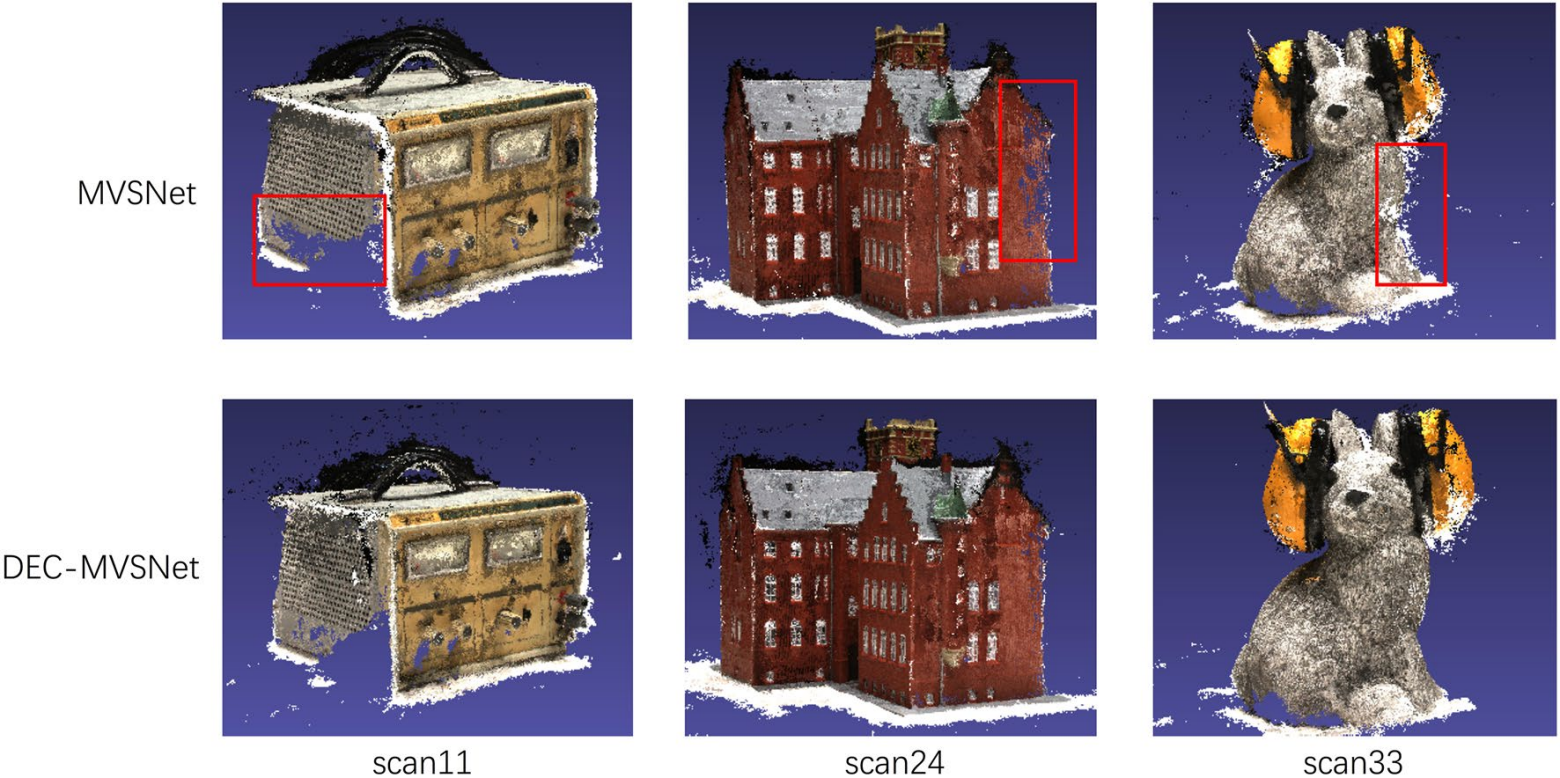
Comparison of the reconstructed point cloud results of Scan11, scan24 and scan33 in the DTU dataset. The red box highlights the difference between the reconstruction results.

In order to verify the performance of our model, the reconstruction results of our model and MVSNet in the DTU dataset are compared, and the results are shown in Fig. 10, which shows that MVSNet reconstructs poorly in areas such as weak texture and has voids in some places. In contrast, the reconstruction results of DEC-MVSNet are better than MVSNet in terms of completeness and overall quality. The GPU memory required for training the networks and the time required to predict per view depth maps are given in Table 3.

**Table 3:** Using the DTU dataset for training, we measured the GPU memory required for training the networks of MVSNet and our model, as well as the time needed to predict depth maps for each view.

Method	GPU Mem (GB)	Runtimes (s)
MVSNet	15.4	1.18
Ours	14.9	2.64

We also compare with SurfaceNet^21^, MVS^22^, MVSNet^11^, R-MVSNet^12^, and P-MVSNet^13^ on the DTU dataset, and the results of the quantitative evaluation are shown in Table 4.

**Table 4:** Quantitative results on the DTU’s evaluation set^18^.

Method	Acc	Comp	Overall
SurfaceNet^21^	0.450	1.040	0.745
MVS^22^	0.760	0.515	0.637
MVSNet^11^	**0.396**	0.527	0.462
R-MVSNet^12^	0.385	0.459	0.422
P-MVSNet^13^	0.406	0.434	0.420
Ours	0.425	**0.411**	**0.418**

We evaluate all methods using both the distance metric^18^ (lower is better).

Significant values are given in bold.

From Table 3, although the additional modules we introduced do not provide a speed advantage, these improvements significantly enhance the model's reconstruction completeness and overall quality. We believe that this trade-off is reasonable for applications requiring high integrity.

In the field of 3D reconstruction, completeness (Comp) and accuracy (Acc) often have a mutually restrictive relationship. As can be seen from Table 4, our model performs well in terms of completeness. Higher completeness means that more point clouds can be reconstructed, which is especially critical in difficult-to-recover areas that are difficult to deal with traditional methods. However, as the number of point clouds increases, especially within these complex regions, it becomes more challenging to guarantee the precise location of each point, so our model fails to achieve optimal accuracy. Nonetheless, in terms of overall quality, our reconstruction results are significantly better than MVSNet.

### Experimental analysis on the BlendedMVS dataset

**Figure 11:**
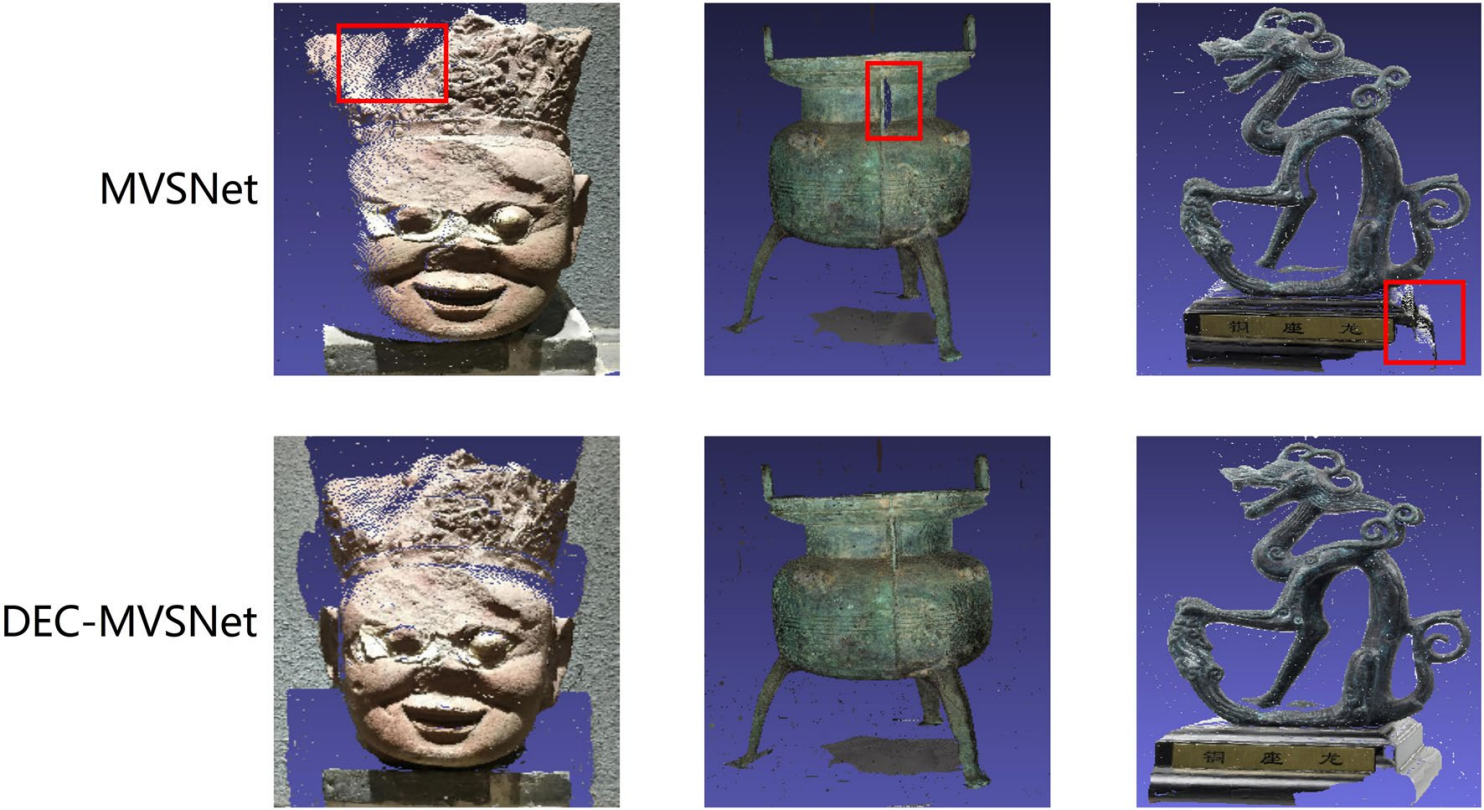
In the BlendedMVS dataset, comparison images of point cloud reconstructions by MVSNet and DEC-MVSNet.

In order to verify the generalization ability of our model DEC-MVSNet, DEC-MVSNet was experimented on the BlendedMVS dataset, and MVSNet and DEC-MVSNet were trained and tested separately in the same environment. In the test metrics, Loss refers to the average L1 error between the predicted and the actual depth maps, with the L1 formula provided in Eq. (10), ave_pre1 denotes the proportion of pixels where the error between the predicted depth map and the real depth map is less than 1 mm, and ave_pre3 denotes the proportion of pixels where the error between the predicted depth map and the real depth map is less than 3 mm. The quantitative evaluation results are shown in Table 5. Figure 11 shows a comparison of the reconstruction results from our model and MVSNet on the BlendedMVS dataset.

10 $${\text{Loss}}({\text{L}}1) = \frac{1}{N}\mathop \sum \limits_{i = 1}^{N} \left| {D_{{pred_{i} }} - D_{{true_{i} }} } \right|$$

where $D_{{pred_{i} }}$ is the pixel value in the predicted depth map,$D_{{true_{i} }}$ is the pixel value in the true depth map, and $N$ is the total number of pixels.

**Table 5:** The evaluation results of MVSNet and DEC-MVSNet models on the BlendedMVS dataset, which use Ave_per1 and Ave_per3 as evaluation indicators, assess the effectiveness of the model.

Method	Loss	Ave_per1	Ave_per3
MVSNet	1.42	0.77	0.91
Ours	**1.35**	**0.82**	**0.96**

Significant values are given in bold.

As can be seen from Table 5, compared to MVSNet, for Loss, the Loss of DEC-MVSNet is reduced from 1.42 to 1.35, in terms of depth map error, our results are better than MVSNet's. From Fig. 11, it is evident that our model also has a clear advantage in the comparison of point cloud data from the reconstruction results and in summary, Through these quantitative results and reconstructed point cloud display, it is proved that the generalization ability of this method DEC-MVSNet is good.

## Conclusion

With the popularization of modern image acquisition devices, the application of 3D reconstruction technology is more and more widely demanded in people's lives. This paper is dedicated to solving the key problems in the field of multi-view 3D reconstruction. Aiming at the problems of general performance in feature extraction and insufficient correlation between cascade structure cost volumes in deep learning-based multiview stereo reconstruction methods. A new end-to-end deep learning network structure with excellent performance, DEC-MVSNet, is proposed. Different from the existing methods, our model optimizes the feature extraction network by incorporating the DE module and residual structure on the basis of the MVSNet framework. By introducing the dilated convolution of the HDC principle and adding the ECA-Net network, the key deep feature information in the image can be effectively extracted, the receptive field of these features can be expanded. Through the residual structure, features can be seamlessly spliced and merged, preserving the global context of the source image while refining intricate details. In the cost volume regularization stage, the CBAM module is introduced to deeply explore the potential relationship between channel and spatial features, enhancing the information connection between multi-scale feature volumes. And further improve the accuracy of the probability volume, thus providing a solid foundation for the subsequent generation of depth maps. Through a series of experimental validations, our DEC-MVSNet has achieved significant effect enhancement on the multi-view 3D reconstruction task.

In our future work, in order to adapt to more complex and higher resolution scenes, reducing the memory and improving the accuracy will become our next main exploration direction.

## Data Availability

The datasets used and/or analyzed during the current study are available under dataset names “DTU”: https://roboimagedata.compute.dtu.dk/. “BlendedMVS”: https://paperswithcode.com/dataset/blendedmvs.
